# Genotype by yield* trait (GYT) biplot analysis: A novel approach for phenotyping sunflower single cross hybrids based on multiple traits

**DOI:** 10.1002/fsn3.3524

**Published:** 2023-06-24

**Authors:** Amir Gholizadeh, Mehdi Ghaffari

**Affiliations:** ^1^ Crop and Horticultural Science Research Department, Golestan Agricultural and Natural Resources Research and Education Center, Agricultural Research Education and Extension Organization (AREEO) Gorgan Iran; ^2^ Oil Crops Research Department, Seed and Plant Improvement Institute Agricultural Research Education and Extension Organization (AREEO) Karaj Iran

**Keywords:** GYT biplot, hybrid, multiple traits, seed yield, sunflower

## Abstract

Sunflower is one of the most important oilseed plants in the world and its oil has nutritional and high economic value. Selection of high‐yielding hybrids is important in sunflower breeding. In this regard, 11 new hybrids along with four cultivars were evaluated in a randomized complete block design with four replications during the 2018–2020 growing seasons. The phenological and agronomic traits including days to flowering, days to ripening, plant height, stem diameter, head diameter, seed number per head, thousand‐seed weight, oil content, and seed yield were measured. In this study, the methods of genotype × trait (GT) and genotype × yield × trait biplot (GYT) were used to identify interrelationships between different traits and to select the best sunflower hybrids based on multiple traits. According to the results, GYT biplot method was more efficient compared to the GT biplot method. Considering both superiority index (SI) and GYT biplot, the genotypes G8, G11, G5, and G3 were superior in terms of agronomical attributes such as flowering and maturity times, stem and head diameter, plant height, thousand‐seed weight, and seed number per head in close relationship with grain yield. Oil content of the hybrids G8, G11, G5, and G3 was 47.9%, 46.4%, 45.8%, and 46.3%, respectively. The results indicated that there is a potential for simultaneous genetic improvement of the characteristics (i.e., plant height, thousand‐seed weight, seed number per head, early maturity) in sunflower. Overall, the GYT graphical biplot method provides a practical and efficient new approach for the identification of suitable hybrids according to the set of intended characteristics in sunflower improvement under multi‐years or multi‐locations.

## Graphical abstract

In this study, the methods of genotype × trait (GT) and genotype × yield × trait biplot (GYT) were used to identify interrelationships between different traits and to select the best sunflower hybrids based on multiple traits. Generally, the graphical method of the GYT biplot provides a practical and efficient new way to identify superior genotypes based on multiple traits in sunflower breeding programs.

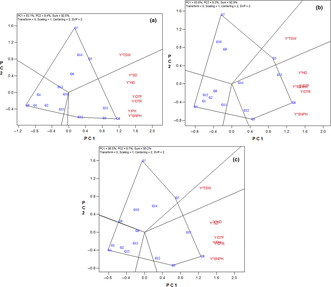

## 1 INTRODUCTION

Sunflower (*Helianthus annuus* L.) is one of the main sources of vegetable oil with an annual production area of about 27 million hectares worldwide (FAO, 2019). Cultivation of oil‐type sunflower in Iran has been started since 1965 and increased to more than 100,000 ha in early 90s (Ghaffari et al., 2020). Sunflower breeding in Iran has been implemented since 1969 and had remarkable achievements in the improvement of sunflower hybrids (Ghaffari et al., 2019). The identification of superior genotypes is very important in the improvement of sunflower. Because of lower heritability and involvement of genotype by environment interaction effects, the selection of superior genotypes in terms of grain yield is complicated (Gholizadeh & Dehghani, 2016, 2017). Beside yield components, agronomic characteristics are relatively heritable, so, indirect selection through other traits is used in breeding programs, which, in turn, helps to increase the breeding cultivars (Mohammadi, 2019).

Different methods are used for studying the relationships among different plant characteristics for use in selection procedure in breeding programs. The genotype by trait (GT) and genotype by yield by trait (GYT) graphical analysis are among these methods that provide graphical and clear images of trait and genotype relationships. The GT biplot analysis has been used for this purpose in some crops such as soybean (Yan & Rajcan, 2002), lupin (Rubio et al., 2004), rapeseed (Dehghani et al., 2008), corn (Santana et al., 2021; Santos et al., 2021; Shojaei et al., 2020), wheat (Zulfiqar et al., 2021), and sorghum (Mukondwa et al., 2021). A potential constraint of the GT biplot method is that it may fail to explain most of the variation and therefore fail to display all patterns of the data and this is most likely to occur with large datasets, small main effects, and complex interactions. Also, the GT biplot method is not able to distinguish the effect of all the traits on yield combination, while the GYT biplot method recently improved to eliminate this deficiency. The GYT biplot method has been introduced as an effective and comprehensive method that graphically identifies the strengths and weaknesses of each genotype and provides a superiority index (SI) for the evaluation of genotypes based on combining all the traits with yield (Kendal, 2019; Yan & Frégeau‐Reid, 2018). This method has been used for evaluation and selection of the genotypes based on multiple traits combined with seed yield in different environments in durum wheat (Kendal, 2019), sesame (Boureima & Abdoua, 2019), barley (Hudzenko et al., 2021), rapeseed (Gholizadeh et al., 2022), and Urochloa sp (Gouveia et al., 2022). As the novelty of this study, so far GYT biplot method had not been used for the evaluation of the relationships between agronomic characteristics and for the selection of genotypes based on multiple traits in sunflower. Hereupon, the objectives of the present study were (1) to investigate the interrelationships among different traits, and (2) to select the preferable sunflower hybrids based on a set of traits affecting grain yield.

## 2 MATERIALS AND METHODS

In this study, 11 new sunflower hybrids along with four commercial hybrids, Golsa, Ghasem, Shams, and Farrokh, were evaluated in a randomized complete block design with four replications at the Seed and Plant Improvement Institute (SPII), Karaj, Alborz Province (50°54′ E longitude, 35°56′ N latitude and 1312 m above mean sea level) for two growing seasons (2018–2019 and 2019–2020). All the hybrids that were used in this study are produced based on cytoplasmic male sterility (PET system + Fertility restorer genes) in Karaj (Seed and Plant Improvement Institute, SPII). Specific mating design has not been used to produce these hybrids. But to evaluate the combing ability of the relevant parent‐inbred lines in the past years, Line × Tester method has been used. All the evaluations were not necessarily done in one experiment and the lines were gradually identified at different times and used in the final crossings to produce the hybrids in this study. The pedigree of the hybrids is presented in Table 1. In that RGKs are restorer lines and AGKs are CMS lines. The experimental plots consisted of three rows each one had three meters in length with 60 cm spacing between rows and 25 cm within rows. The phenological and agronomic traits including days to flowering (DTF), days to ripening (DTR), plant height (PH), stem diameter (SD), head diameter (HD), seed number per head (SNPH), thousand‐seed weight (TSW), oil content (OC), and seed yield (SY) were measured. Average of measured traits in 15 sunflower hybrids in two experimental years is displayed in Table 2.

**TABLE 1:** Genotypic code, name, and origin of the tested sunflower hybrids.

No	Code	Name/pedigree	Origin
1	G1	RGK25 × AGK330	Iran
2	G2	RGK15 × AGK376	Iran
3	G3	RGK15 × AGK370	Iran
4	G4	RGK15 × AGK358	Iran
5	G5	RGK111 × AGK32	Iran
6	G6	RGK21 × AGK2	Iran
7	G7	RGKo54 × AGKo60	Iran
8	G8	RGK15 × AGK1221	Iran
9	G9	RGK21 × AGKo42	Iran
10	G10	RGK111 × AGK78	Iran
11	G11	RGK24 × AGK370	Iran
12	G12	Golsa	Iran
13	G13	Ghasem	Iran
14	G14	Shams	Iran
15	G15	Farrokh	Iran

### 2.1 Statistical analysis

#### 2.1.1 Analysis of variance

In order to test the normality of the data, the Shapiro–Wilk test was used by SPSS (Ver. 19) software (SPSS, 2010). Combined analysis of variance was used to separate variance components using general linear model (GLM) procedure in Statistical Analysis System (SAS) (SAS, 2003), wherein genotype and year were considered as the fixed and random effects, respectively.

#### 2.1.2 Biplot methodologies

The biplots for GT were generated by the first two principal components obtained from singular value decomposition (SVD) using GGEbiplot software (Yan & Kang, 2002; Yan & Rajcan, 2002). The results of this analysis were used to study the interrelationships between agronomic characteristics and to identify suitable sunflower hybrids based on a set of desired traits.

The GYT biplots were provided according to the method of Yan and Frégeau‐Reid (2018) for multi‐traits data of genotypes in single and multi‐year trials using GGE‐biplot software, where the weight of each attribute is determined by dividing or multiplying by the grain yield. For attributes for which a large numerical value is desirable as stem and head diameter, seed number per head, and seed weight, the numerical values for each characteristic were multiplied by grain yield. While for the attributes for which a small numerical value is desirable as plant height and days to flowering and ripening, the values for each trait were divided by the grain yield. The superiority index (SI) was estimated according to the standardized GYT for all combinations of yield by traits (Yan & Frégeau‐Reid, 2018). The results of GYT biplot were used to study the interrelationships between agronomic traits and to identify desirable sunflower hybrids according to the yield by trait combinations.

## 3 RESULTS

### 3.1 Analysis of variance

The results of the combined analysis of variance showed that the effect of the year (Y) was significant for days to flowering, head diameter, stem diameter, and thousand‐seed weight (Table 3). The effect of genotype (G) was significant for all studied traits. The G × Y interaction was also significant for all traits except days to ripening, seed number per head, and seed yield (Table 3). The lowest and highest coefficients of variation were 1.72% and 13.14% for days to flowering and seed number per head, respectively (Table 3).

**TABLE 2:** Average of measured traits in 15 sunflower hybrids in two experimental years.

Genotype	DTF	DTR	PH (cm)	HD (cm)	SD (mm)	TSW (g)	SNPH	OC (%)	SY (kg h^−1^)
G1	59	105	190.38	20.00	25.33	56.33	757.13	43.3	2556
G2	55	101	186.00	18.13	25.44	58.35	769.13	44.2	2677
G3	55	100	173.88	18.50	23.37	57.20	1017.75	46.3	3444
G4	57	99	181.25	19.44	25.76	56.60	733.25	42.9	2459
G5	58	110	180.75	20.50	25.31	69.30	837.63	45.8	3501
G6	58	102	166.13	20.88	24.63	63.05	753.00	44.6	2847
G7	55	105	180.00	20.38	24.58	85.98	570.75	46.1	2927
G8	55	104	160.88	19.94	22.54	60.33	1064.25	47.9	3836
G9	56	104	172.13	20.41	23.83	66.90	646.00	44.2	2625
G10	57	106	175.13	18.69	22.07	71.68	687.75	43.2	2930
G11	58	105	159.25	19.88	24.73	63.78	948.63	46.4	3605
G12	56	101	162.88	18.25	22.89	58.73	907.50	45.5	3205
G13	54	102	169.63	18.75	23.15	61.45	779.25	46.0	2864
G14	59	108	190.38	19.50	24.85	68.15	785.88	44.8	3209
G15	56	103	166.38	19.25	20.35	59.63	779.75	43.1	2786
LSD 5%	1.0	1.9	14.1	1.6	1.1	5.0	104.8	1.0	304.5
LSD 1%	1.3	2.6	18.7	2.1	1.5	6.6	138.9	1.4	403.6

Abbreviations: DTF, days to flowering; DTR, days to ripening; HD, head diameter; OC, oil content; PH, plant height; SD, stem diameter; SNPH, seed number per head; SY, seed yield; TSW, thousand‐seed weight.

### 3.2 The GT biplot for grouping the hybrids

**FIGURE 1:**
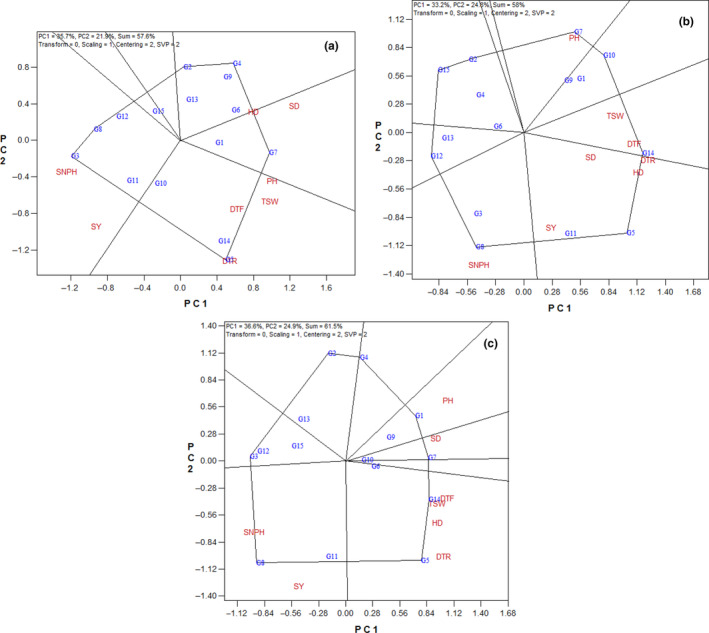
Polygon view of the genotype × trait biplot of sunflower hybrids. (a) First year, (b) second year, (c) average of 2 years. DTF, days to flowering; DTR, days to ripening; HD, head diameter; PH, plant height; SD, stem diameter; SNPH, seed number per head; SY, seed yield; TSW, thousand‐seed weight. Refer Table 1 for genotype name.

According to the results of GT‐biplot analysis, the first two principal components accounted for 35.7% and 21.9% variability of standardized data, respectively (in total 57.6%), in the first year (Figure 1a). The polygon view is one of the most important applications of GT biplot to identify the hybrids that have the highest value for one or more traits. The vertex hybrid in each division in the polygon view is the superior hybrid regarding the test trait(s). According to Figure 1a, the vertex hybrids were G3, G8, G2, G4, G7, and G5. The hybrids G3 and G8 were the most favorable hybrids for seed yield and seed number per head (Figure 1a). The hybrid G7 had the highest values for stem diameter and head diameter. Also, the hybrid G5 has the highest values for days to flowering, days to ripening, plant height, and thousand‐seed weight. On the other hand, the vertex hybrids G2 and G4 were not suitable for any of the measured traits (Figure 1a).

In the second year, the GT biplot for the dataset explained 58% (33.2% and 24.8% by PC1 and PC2, respectively) of the total variation of the standardized data (Figure 1b). According to Figure 1b, there are seven vertex hybrids in the second year (Figure 1b) which are hybrids G12, G15, G2, G7, G10, G14, and G5. The hybrid G8 had the highest values of seed number per head. Also, hybrid G5 was the favorable hybrid for days to ripening, stem diameter, head diameter, and seed yield (Figure 1b). The vertex hybrid G14 was the most favorable hybrid for days to flowering, and thousand‐seed weight (Figure 1b). Also, vertex hybrid G7 had the highest values of plant height. The vertex hybrids G12, G15, G2, and G10 were not suitable for any of the measured traits (Figure 1b).

Results of GT biplot analysis for an average of 2 years indicated that the first two principal components explained 36.6% and 24.9%, variation of the standardized data, respectively (in total 61.5%) (Figure 1c). According to Figure 1c, the vertex hybrids were G8, G3, G2, G4, G1, G7, G14, and G5. The hybrid G8 had the highest values for seed yield and seed number per head. The hybrids G5 and G14 were the most favorable hybrids for days to flowering, days to ripening, head diameter, and thousand‐seed weight (Figure 1c). The hybrid G1 had the highest values of plant height. Also, the vertex hybrid G7 was the most favorable hybrid for stem diameter. On the other hand, the vertex hybrids G3, G2, and G4 were not suitable for any of the understudy traits in the average 2 years (Figure 1c).

### 3.3 The GT biplot for displaying the relationships among the traits

**FIGURE 2:**
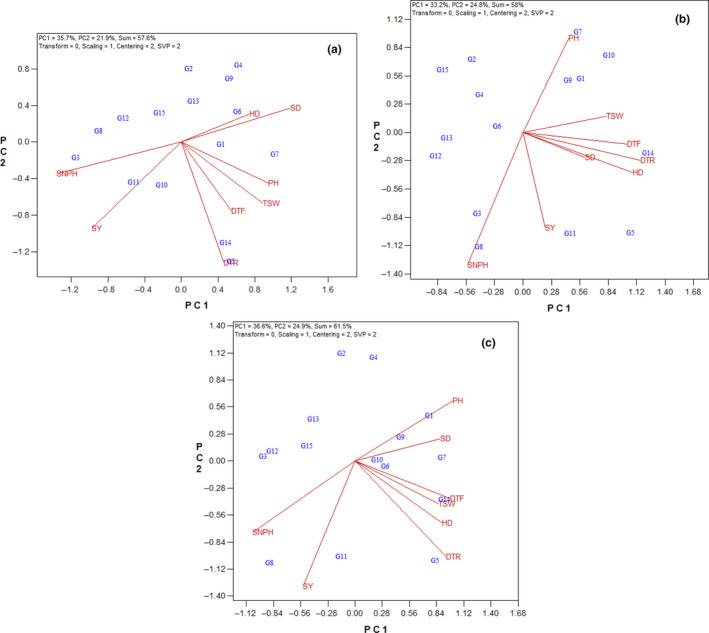
Vector view of the genotype × trait biplot of sunflower hybrids. (a) First year, (b) second year, (c) average of 2 years. DTF, days to flowering; DTR, days to ripening; HD, head diameter; PH, plant height; SD, stem diameter; SNPH, seed number per head; SY, seed yield; TSW, thousand‐seed weight.

The vector view is one of the applications of the GT biplot to examine interrelationships between environments. In the vector view of the GT biplot, the lines that connect each trait to the origin are called the vector. The angle between the vectors is an indicator of the relationship between the traits. The angles lower and more than 90° imply positive and negative correlations, respectively. Also, if the angle is near 90°, it showed no relationship. According to Figure 2a in the first year, there were high positive correlations between seed number per head and seed yield, between stem diameter and head diameter, and between days to flowering, days to ripening, plant height, and thousand‐seed weight.

The vector view in the second year (Figure 2b) indicated that there were high positive correlations between seed yield with seed number per head. Also, Figure 2b shows that there was a positive correlation between days to flowering, days to ripening, stem diameter, head diameter, and thousand‐seed weight. Results of the vector view for an average of 2 years indicated also that there were high positive correlations between seed yield with seed number per head (Figure 2c). The correlations between stem diameter with plant height on one hand and days to flowering and ripening and head diameter with seed weight on the other hand were highly positive (Figure 2c).

### 3.4 The GYT biplot for classification of the hybrids

**FIGURE 3:**
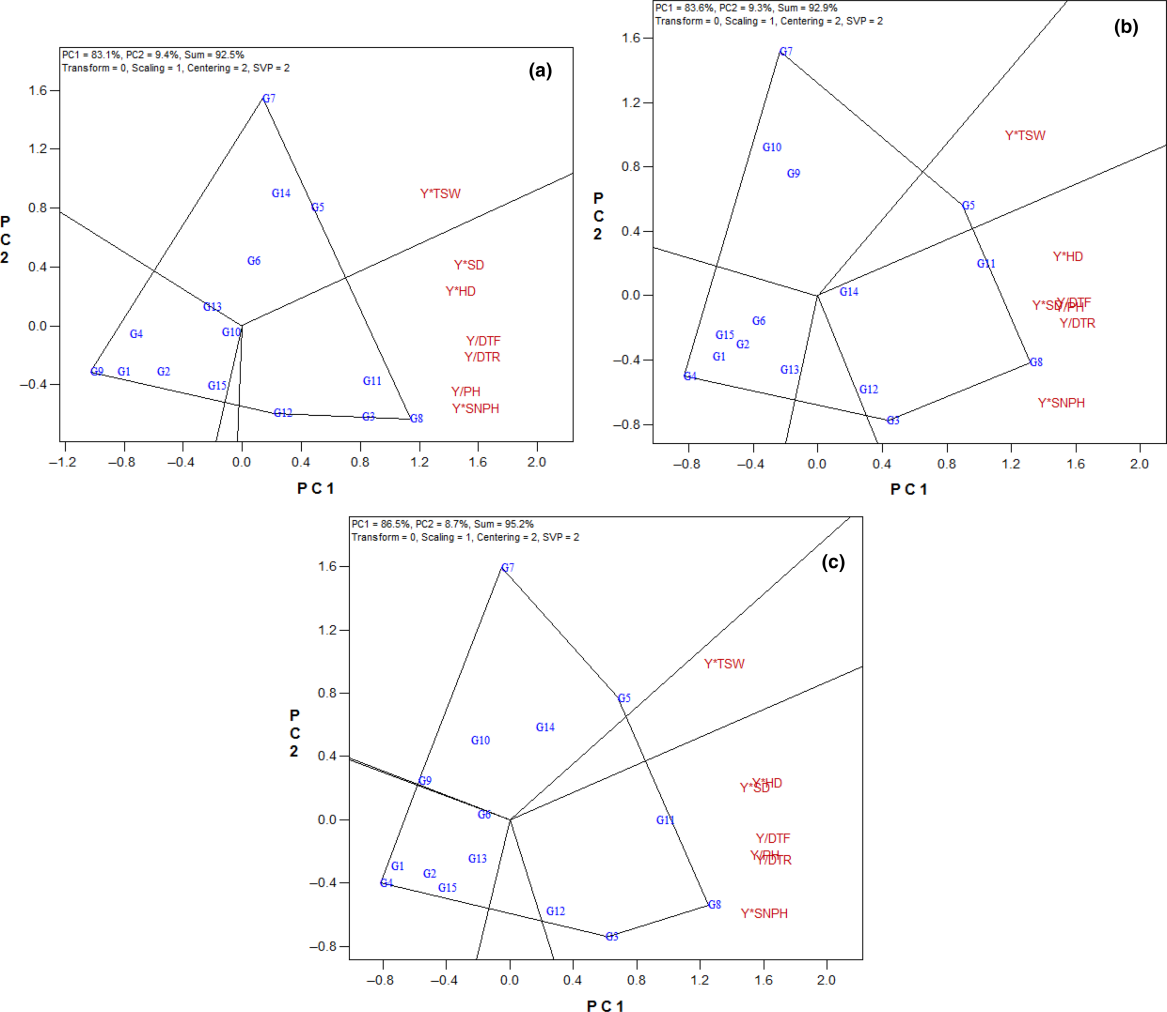
Polygon view of the genotype × yield × trait biplot of sunflower hybrids. (a) First year, (b) second year, (c) average of 2 years. DTF, days to flowering; DTR, days to ripening; HD, head diameter; PH, plant height; SD, stem diameter; SNPH, seed number per head; TSW, thousand‐seed weight; Y, seed yield. Refer Table 1 for genotype name.

In the first, second, and average of 2 years, breeding hybrids G8, G3, G12, and G11 had the highest values for Y*SNPH, Y*PH, Y/DTR, Y/DTF, Y*HD, and Y*SD in polygon view of the GYT biplot (Figure 3), which implies the superiority of these hybrids when considering the combination of seed yield with days to flowering and ripening, plant height, stem and head diameter, and seed number per head. The breeding hybrids G5 and G7 with the high value of Y*TSW were the best in combining seed yield with a number of thousand‐seed weight.

### 3.5 The GYT biplot for representation of the yield–trait relationships

**FIGURE 4:**
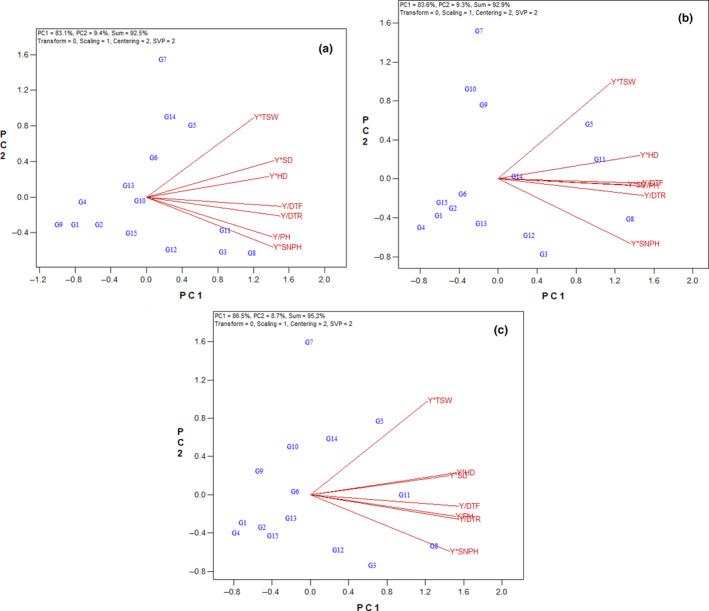
Vector view of the genotype × yield × trait biplot of sunflower hybrids. (a) First year, (b) second year, (c) average of 2 years. DTF, days to flowering; DTR, days to ripening; HD, head diameter; PH, plant height; SD, stem diameter; SNPH, seed number per head; TSW, thousand‐seed weight; Y, seed yield.

Based on the states of the vectors of the GYT biplot, considerable correlations were observed between Y*SD with Y*HD; Y/DTF with Y/DTR; and Y*PH with Y*SNPH, in the first year as expressed by acute angles between the related vectors (Figure 4a). In the second year, there were higher correlations between Y*SD, Y*HD, Y*PH, and Y/DTF with Y/DM (Figure 4b). Also, based on average of 2 years, the most remarkable positive correlations were between Y*SD with Y*HD; and between Y*PH, Y/DTF with Y/DTR (Figure 4c).

### 3.6 Superiority vs. “weaknesses and strengths” of hybrids

**FIGURE 5:**
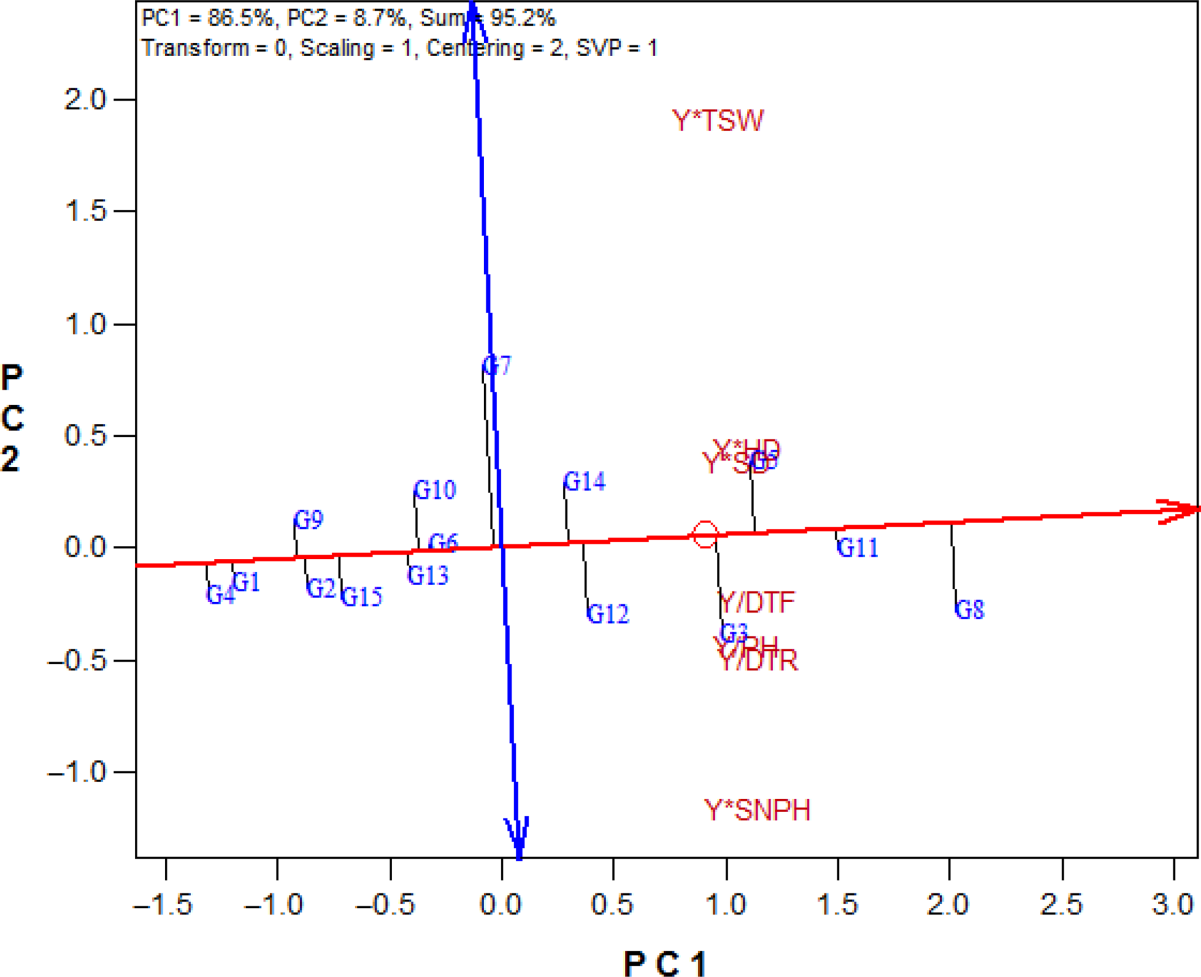
Average tester coordinate (ATC) view of the genotype × yield × trait biplot to rank the genotypes based on overall superiority and their strengths and weaknesses. DTF, days to flowering; DTR, days to ripening; HD, head diameter; PH, plant height; SD, stem diameter; SNPH, seed number per head; TSW, thousand‐seed weight; Y, seed yield. Refer Table 1 for genotype name.

The expression of the GYT biplot via average tester coordinate (ATC) indicated that, regarding all of the yield–trait combinations, hybrids G8, G11, G5, and G3 were superior to four commercial hybrids and others hybrids, while the hybrids G4, G1, G9, G2, and G15 were unfavorable (Figure 5). Using the superiority index (SI), the understudied hybrids were ranked based on all of the yield by trait combinations from the most favorable to the most unfavorable was as follows: G8 > G11 > G5 > G3 > G12 > G14 > G7 > G6 > G10 > G13 > G15 > G2 > G9 > G1 > G4 (Table 4). Oil content of the hybrids G8, G11, G5, and G3 was 47.9%, 46.4%, 45.8%, and 46.3%, respectively (Table 2).

**TABLE 3:** Combined analysis of variance for seed yield of sunflower hybrids.

Source of variations	df	Mean square							
		DTF	DTR	PH	HD	SD	TSW	SNPH	SY
Year (Y)	1	35.21**	35.21^ns^	26.13^ns^	30.00*	265.61**	2902.80**	282,949.41^ns^	20,901,722.70^ns^
Replication/Y	6	1.52	52.33	1975.38	4.56	3.85	18.37	390,247.68	5,239,126.91
Genotype (G)	14	19.91**	63.88**	840.24**	6.19**	18.37**	488.48**	143,758.79**	1,380,841.80**
G × Y	14	2.74**	1.64^ns^	563.26**	11.09**	11.86**	111.36**	11,417.62^ns^	129,824.50^ns^
Error	84	0.94	3.76	200.71	2.65	1.33	25.07	11,117.60	93,801.54
CV (%)		1.72	1.87	8.13	8.29	4.82	7.84	13.14	10.10

*Note*: ns, * and ** nonsignificant and significant at the 0.05 and 0.01 probability level, respectively.

Abbreviations: DTF, days to flowering; DTR, days to ripening; HD, head diameter; PH, plant height; SD, stem diameter; SNPH, seed number per head; SY, seed yield; TSW, thousand‐seed weight.

**TABLE 4:** Standardized genotype × yield × trait (GYT) values in 15 sunflower hybrids in two experimental years.

Genotype	Y/DTF	Y/DTR	Y/PH	Y*HD	Y*SD	Y*TSW	Y*SNPH	SI
G1	−1.41	−1.24	−1.55	−0.95	−0.77	−1.44	−0.76	−1.16
G2	−0.69	−0.67	−1.22	−1.26	−0.44	−1.09	−0.58	−0.85
G3	1.21	1.46	0.61	0.54	0.82	0.09	1.45	0.88
G4	−1.45	−1.15	−1.50	−1.34	−0.92	−1.58	−0.94	−1.27
G5	0.86	0.80	0.46	1.50	1.64	1.42	0.65	1.05
G6	−0.61	−0.31	−0.29	0.04	−0.23	−0.41	−0.46	−0.33
G7	−0.07	−0.29	−0.59	0.06	−0.05	1.68	−1.13	−0.06
G8	2.18	2.15	1.98	2.06	1.42	1.09	2.26	1.88
G9	−1.01	−1.01	−0.93	−0.66	−1.00	−0.52	−1.09	−0.89
G10	−0.35	−0.34	−0.43	−0.52	−0.78	0.47	−0.64	−0.37
G11	1.16	1.45	1.57	1.49	1.69	1.05	1.33	1.39
G12	0.49	0.75	0.57	−0.07	0.10	−0.16	0.61	0.33
G13	−0.18	−0.28	−0.38	−0.64	−0.62	−0.51	−0.34	−0.42
G14	0.02	0.20	−0.39	0.41	0.74	0.72	0.07	0.25
G15	−0.55	−0.54	−0.43	−0.65	−1.59	−0.80	−0.42	−0.71

Abbreviations: DTF, days to flowering; DTR, days to ripening; HD, head diameter; PH, plant height; SD, stem diameter; SI: superiority index; SNPH, seed number per head; TSW, thousand‐seed weight; Y, seed yield.

## 4 DISCUSSION

The cultivation and breeding of oilseeds to increase yield are necessary for Iran because the amount of demand is greater than the amount of production. Among the oil crops, sunflower as a spring plant, it has great adaptability to different geographical regions of Iran (Ghaffari et al., 2020, 2021). Therefore, it is very important to improve high‐yielding cultivars suitable for these areas. There are always two main problems in introducing new varieties. The expression of genotype by environment interaction (GEI) effect is the first and most important one. Through the evaluation of new cultivars in the target areas, the adaptability of the new genotypes in these areas is evaluated and the issue is managed as it is possible. The second case is the complexity of breeding cultivars that are superior in terms of other agricultural characteristics in addition to grain yield. Previous experiences show that due to the low heritability of seed yield, it is necessary to consider other agronomic characteristics (morphological, agronomical, and physiological traits) in the selection process of desirable genotypes.

Various methods have been introduced for the simultaneous selection of seed yield along with other agronomic traits. Among them are GT and GYT biplot methods that could provide graphic images and overview of the original data. In comparison with conventional methods such as correlation coefficients or multivariate, GT and GYT biplot methods are efficient statistical facilities for visual evaluation, classification, and selection of suitable genotypes (Gholizadeh & Dehghani, 2016). The GT‐biplot method has been used to classify and evaluate genotypes and to identify the important traits affecting grain yield in other crops (Malik et al., 2014; Santana et al., 2021; Santos et al., 2021; Yan & Rajcan, 2002); however, this method cannot identify the combined effect of all other traits on grain yield. Instead, GYT biplot methodology that has been introduced recently solved the defect of GT method and is able to identify the combined effect of other traits on grain yield. This methodology providing a superiority index (SI) is an effective method that graphically discovers the positive and negative aspects of a given genotype in terms of all yield‐affecting traits (Kendal, 2019; Yan & Frégeau‐Reid, 2018). This method has not been used previously in sunflower selection programs and is considered as an innovative aspect of this study.

The results of this study indicate that method GYT approach is more efficient in sunflower selection programs compared to GT methodology. According to the results of this study and considering the combined effect of investigated traits, i.e., days to flowering, days to ripening, height of plant, stem and head girth, seed number per head, and seed weight on grain yield, the breeding hybrids G8, G11, G5, and G3 were superior genotypes in this study. In contrast, G4, G1, G9, G2, and G15 were ranked the poorest. In fact, selection based on a set of useful traits increases the agricultural value of a cultivar, and therefore, the hybrids G8, G11, G5, and G3 are superior hybrids that are recommended for growing in target locations. The GYT biplot approach in this study revealed the positive correlation of yield–trait combinations which is an important feature of this method compared with the GT biplot method. This feature can lead to the reduction of evaluated traits in the selection process of superior genotypes and reduce the cost of evaluation experiments in sunflower breeding projects. Accordingly, due to the high correlation of stem diameter with head diameter one of these traits (i.e., head diameter) can be used as the selection criterion and so on because of high correlation of days to flowering, days to ripening, and plant height, the simplest one (i.e., days to flowering) could be used as suitable selection criterion.

## 5 CONCLUSION

The unique feature of this study is that it showed that GYT biplot method can be used as a comprehensive and effective method for the evaluation and selection of sunflower single cross hybrids based on a set of different agronomical traits under multi‐years or multi‐locations. The results showed that it is possible to select hybrids based on multiple traits for improving genetic material in sunflower breeding programs. The breeding hybrids G8, G11, G5, and G3 were superior regarding the relationships of all the studied traits with sunflower grain yield, which implies an increase in selection efficiency. Oil content of the hybrids G8, G11, G5, and G3 was 47.9%, 46.4%, 45.8%, and 46.3%, respectively. The results also indicate that simultaneous improvement of some of agronomic characteristics such as early maturity, plant height, stem, and head diameter is possible in sunflower breeding programs.

## AUTHOR CONTRIBUTIONS

**Amir Gholizadeh:** Formal analysis (lead); investigation (equal); methodology (equal); resources (lead); software (lead); validation (equal); writing – original draft (lead); writing – review and editing (equal). **Mehdi Ghaffari:** Conceptualization (lead); data curation (lead); funding acquisition (lead); investigation (equal); methodology (equal); project administration (lead); supervision (lead); visualization (lead); writing – review and editing (equal).

## CONFLICT OF INTEREST STATEMENT

The authors declare that they have no conflict of interest.

## ETHICS STATEMENT

This study does not involve any human or animal testing.

## Data Availability

The data that support the findings of this study are available from the corresponding author upon reasonable request.
